# Clinical outcomes associated with albuminuria in central Australia: a cohort study

**DOI:** 10.1186/s12882-016-0328-1

**Published:** 2016-08-05

**Authors:** Rebecca Ritte, Joanne Luke, Craig Nelson, Alex Brown, Kerin O’Dea, Alicia Jenkins, James D. Best, Robyn McDermott, Mark Daniel, Kevin Rowley

**Affiliations:** 1 Onemda Group, Indigenous Health Equity Unit, Centre for Health Equity, Melbourne School of Population and Global Health, The University of Melbourne, VIC Melbourne, 3010 Australia; 2 Western Health, Footscray, VIC 3011 Australia; 3 Northwest Academic Centre, The University of Melbourne, Melbourne, VIC 3010 Australia; 4 South Australian Health and Medical Research Institute, Adelaide, SA 5000 Australia; 5 Melbourne School of Population and Global Health, The University of Melbourne, Melbourne, VIC 3010 Australia; 6 NHMRC Clinical Trials Centre, University of Sydney, Camperdown, NSW 1450 Australia; 7 Faculty of Medicine Dentistry and Health Sciences, The University of Melbourne, Melbourne, VIC 3010 Australia; 8 Lee Kong Chian School of Medicine, Imperial College London and Nanyang Technological University, Singapore, Singapore; 9 Centre for Chronic Disease Prevention, James Cook University, Cairns, QLD 4870 Australia; 10 School of Population Health, University of South Australia, Adelaide, SA 5000 Australia

**Keywords:** Aboriginal people, Albuminuria, Albumin creatinine ratio, Risk, Cohort study, End stage renal disease, Rural and remote health

## Abstract

**Background:**

Chronic kidney disease (CKD) and end-stage-kidney disease (ESKD) continue to be under-diagnosed and a major burden for Aboriginal communities in central Australia. The aim of this study was to examine the risk of poor clinical outcomes associated with elevated albumin-to-creatinine ratio (ACR) among Aboriginal people in central Australia.

**Methods:**

Cox proportional hazards models were used to estimate the risk of end stage kidney disease (ESKD), dialysis, CVD (cardiovascular disease) and mortality associated with participants’ baseline albuminuria reading from a 10-year cohort study of Aboriginal people (*n* = 623) from three communities in central Australia. Predictors of progression of albuminuria were also examined in the context of the Kidney Health Australia (KHA) Risk Matrix.

**Results:**

A baseline ACR level of ≥3.5 mg/mmol was associated with an almost 10-fold increased risk of ESKD (95%CI 2.07-43.8) and a 15-fold risk of dialysis (95%CI 1.89-121). Albuminuria ≥3.5 mg/mmol was also associated with a borderline 63 % increased risk of CVD (95%CI 0.98-2.71). No significant association was observed with mortality from all-causes or chronic disease. Diabetes and a waist-to-hip ratio ≥0.90 independently predicted a two-fold increased risk of a progression to higher ACR levels.

**Conclusions:**

A single measure of moderately increased albuminuria was a strong predictor of renal failure in this population. A single spot urine ACR analysis in conjunction with the KHA Risk Matrix may be a useful and efficient strategy to screen for risk of CKD and progression to dialysis in remote communities. A focus on individuals with diabetes and/or central obesity for strategies to avoid increases in albuminuria may also prevent future CKD and CVD complications.

**Electronic supplementary material:**

The online version of this article (doi:10.1186/s12882-016-0328-1) contains supplementary material, which is available to authorized users.

## Background

Recent social history has resulted in chronic kidney disease (CKD) and end-stage-kidney disease (ESKD) among Aboriginal and Torres Strait Islander communities occurring at disproportionately high rates [1, 2] and has been described as an ‘epidemic’ [3]. Risk factors for CKD are likely to be established prior to birth and include environmental and economic determinants arising from invasion and colonisation, and the resulting prevalence of low birthweight/nephron endowment, persistent infections, obesity, hypertension, type-2 diabetes, and the “Westernisation” of diets [4, 5]. These antecedent factors also coincide with the increased risk of other chronic diseases such as cardiovascular disease (CVD) and as these multiple factors accumulate across a person’s life course, a very high risk for chronic disease is created [2, 5]. Furthermore, Aboriginal people have a multiplicity of barriers, including geographical barriers, in receiving efficacious treatment for ESKD [6]; are more likely to be referred late for care; there is often a need for relocation to urban centres; and Aboriginal people are less likely than non-Aboriginal people to receive a kidney transplant [1, 6, 7]. As a result of CKD, communities experience strong negative impacts from the loss of community members who must relocate for treatment, premature death and increased health care costs for primary health care services [6, 8, 9]. Early symptoms of CKD are often silent [8, 10, 11] and little is known about the burden of early CKD for Aboriginal people [1] or of indicators of who progresses to ESKD. The prevalence of preventable chronic diseases, including CKD, have been found to be significantly underestimated, particularly in remote Australian communities, thus highlighting gaps in diagnosis and treatment of CKD [12].

Albuminuria and glomerular function are well-known markers for kidney decline and function [11] however research into the association of albuminuria and its predictive value for ESKD among Aboriginal and Torres Strait Islander populations in Australia is limited [2]. Kidney Health Australia (KHA) has developed a risk matrix to guide clinical CKD management, such as future checks and recommended clinical actions, according to stage of albuminuria and glomerular function [11] based on prospective population health outcome data [13]. A CKD diagnosis involves either two abnormal estimated glomerular filtration rate (eGFR) measurements at least three months apart or two abnormal albumin/creatinine ratio (ACR mg/mmol) measurements at least three months apart [11, 14, 15]. The KHA Risk Matrix is incorporated in the Australian recommendations for CKD diagnosis and management and has also been integrated in the latest edition of the Central Australian Rural Practitioners Association (CARPA) Standard Treatment guidelines [15]. However, a non-invasive and simpler method applicable to the remote setting for screening to identify future CKD risk is required. The single spot urine ACR measurement is included in the Medicare Adult Health Check for Aboriginal and Torres Strait Islander adults [11] and could be a candidate screening tool for assessing future ESKD risk. It is a simple and cost effective method accepted among Aboriginal Health Workers and Aboriginal health service users in remote areas [9] and is amenable to point of care testing [16]. Such a screening tool could trigger preventive strategies for CKD progression including use of appropriate pharmacological and non-pharmacological interventions [4, 15] before a clinical diagnosis of CKD.

Using data from a longitudinal cohort study in central Australia, this study aimed to document the outcomes of renal disease, CVD and mortality according to a single baseline assessment of albuminuria. It aimed to evaluate the applicability of using a single ACR measurement as a screening tool for identifying CKD risk in the primary health care setting in central Australia. Further, given that chronic kidney disease (CKD) and end-stage-kidney disease (ESKD) continue to be under-diagnosed and a major public health burden for Aboriginal in remote communities, this study aimed to identify possible candidates for further screening and CKD prevention strategies by following the progression of participants to worsening albuminuria according the risk categories described in the KHA Risk Matrix (green (normal risk), yellow (mildly increased risk) and red (very high increased risk)) [11]. As a result, this study aimed to have real and practical screening implications for primary health care clinics in remote settings in identifying Aboriginal individuals at elevated risk of CKD and progression to dialysis.

## Methods

The central Australian CVD cohort study is longitudinal retrospective cohort study designed to assess CVD risk factors in three remote central Australian communities [17–19]. The details of recruitment, measurement of baseline variables and biological sample collection in 1995 have been previously reported [17–19]. Participation was voluntary and response rates were 68-85 % of the eligible adult population and 738 persons form the current cohort. As culturally appropriate and for logistical reasons, ACR in early morning urine samples were used as a marker of albumin excretion rate rather than 24 h specimens [18]. Urinary albumin concentration was measured using immunonephelometer (Kallestadt QM300 or Beckman 360 Array nephelometers; interassay coffecient of variance [CV] 3-5 %). Urinary creatinine concentration was measured using an alkaline picrate method (Olympus AU800 autoanalyser; interassay CV 2 %). For persons with urinary albumin below the detectable limit, a pragmatic albumin value of 0.1 mg/L was used in the calculation of ACR.

As previously described [17, 19], for the subsequent follow-up period (from baseline survey in 1995 to 31 December 2004 in two communities and 31 December 2005 in one community), follow-up of the participants’ records was accessed from hospital admissions (including emergency presentations) and primary health care (PHC) care providers to seek information including: ACR; body-mass-index (BMI) and waist-hip-ratio (WHR); smoking status; cardiovascular disease outcomes (clinically diagnosed ischaemic heart disease, ischaemic stroke or peripheral vascular disease (International classification of diseases, 10th revision [ICD-10] codes I20–I25, I63–I69, I70– I89; or 9th revision [ICD-9] codes 410–414, 433–438, 440–459); diabetes (ICD-10 codes E10–E15 or ICD-9 code 250, two or more blood glucose readings >11.1 mmol/L, or two or more fasting blood glucose readings >7.0 or prescription of hypoglycaemic medication); and hypertension (ICD-10 codes I10–I15 or ICD-9 codes 401–405, three or more blood pressure readings >140/90 mmHg, or prescription of antihypertensive medication). Further information was collected from death certificates, autopsy findings and the National Death Index. Primary ESKD was considered on the basis of clinical diagnosis in the participant’s medical records (ICD-10 code N18.6 or ICD-9 code 585.6), an indication of initiating renal replacement therapy or requiring a kidney transplant. For the purpose of this analysis, dialysis for acute kidney failure was not included. Renal mortality was defined as any primary or secondary cause of death associated with renal failure (excluding infections). CVD was considered present with the clinical diagnosis of coronary heart disease (ischaemic heart disease, acute myocardial infarction or angina pectoris [excluding congenital or rheumatic heart diseases]), stroke (ischaemic, haemorrhagic or unspecified), peripheral vascular disease or chronic heart failure indicated by confirmed diagnoses in PHC records, relevant ICD codes in hospital records or cause of death [17, 19]. Date of first diagnosis of each condition was noted, along with date of diagnosis of diabetes.

The present study is based on data from women and men aged 15 years and over. We excluded participants from the main cohort who were diagnosed with CVD (*n* = 89) and/or CKD (*n* = 26) before baseline recruitment. Thus, the number of the cohort participants included in this set of analyses was 623 women and men.

Indirectly standardised for age and sex, incidence rates (with 95 % confidence intervals; CIs) were calculated for outcomes including ESKD, dialysis, CVD, renal mortality, chronic disease mortality (CVD, renal, cancer or diabetic cause of death) and all-cause mortality (including and excluding trauma such as motor vehicle accidents) among all participants and among participants who had a normal (ACR <3.5 mg/mmol) or an abnormal ACR measurement (ACR ≥3.5 mg/mmol). Gender-specific ACR criteria for defining levels of albuminuria were not applied due to the body composition characteristics for this population [18], indeed gender specific cut-offs are not incorporated in the guidelines developed by Kidney Disease Improving Global Outcomes (KDIGO) [20]. A non-diabetic subgroup from the three central Australian communities was used as a reference population for indirect standardisation.

Cox’s proportional hazards models were used to estimate hazard ratios (HR) and 95 % CIs of ESKD, dialysis, CVD, combined chronic disease mortality and all-cause mortality associated with an abnormal baseline albuminuria measurement. The small number of participants (*n* = 51) and follow-up time (663 person-years) did not allow for the estimation of a third category of baseline ACR levels >35 mg/mmol. Person-years were calculated from the time of recruitment until incident diagnosis or end of follow-up period. Multivariable models adjusted firstly for age, gender and then community were assessed. An extended multivariable model with further adjustment for potential confounders was also assessed. Only statistically significant covariates (identified initially through univariate analyses) were assessed using backwards selection and retained in the model. Additional covariates beyond age, gender and community that were assessed included baseline categorical measures of diabetes (yes/no), fasting glucose tertiles, BMI (WHO categories), waist-to-hip ratio (0.90 > WHR < 0.90), abstinence from alcohol (yes/no), current smoking (yes/no), hypertension (SBP ≥140 mmHg and/or DBP ≥90 mmHg or current anti-hypertensive medication including angiotensin-converting-enzyme inhibitor (ACEi)] (yes/no), prevalent UTI (yes/no), haematuria and homocysteine quartiles (μmol/L). Missing values (generally <4 %) were accounted for by creating an extra category in each covariate. A sensitivity analysis was completed to assess the differing influence fasting glucose and baseline diabetes had on the final model. As a result baseline diabetes did not have a strong influence on the final model and was not used in the final model (data not shown).

To assess clinical factors associated with an increase in ACR into categories of moderately increased albuminuria (3.5-35 mg/mmol) and/or severely increased albuminuria (ACR >35 mg/mmol), and therefore increasing in the risk categories of the KHA Risk Matrix, a subgroup of the cohort (*n* = 277) was created among participants who had both a baseline measurement of ACR of ≤35 mg/mmol and a follow-up measurement of ACR in their medical records. Due to the renoprotective association of ACEi and ARBs with CKD we also excluded participants who were taking anti-hypertensive medication (including ACEi and ARBs) [21]. A new outcome event was created to indicate a change in ACR levels that would move the participant to a lower or higher risk category on the KHA Risk Matrix (from green/normal risk category [<3.5 mg/mmol] to yellow/mild risk category [3.5-35 mg/mmol] or red/high risk category [>35 mg/mmol]; or from yellow to red) according to their follow-up ACR measurement. In this analysis, only ACR was used as an indicator of kidney damage. Kidney function (i.e. eGFR) was not used because only 2 participants from the analytical subcohort had a baseline eGFR assessment. We were therefore unable to incorporate the orange/moderate risk category (<3.5 mg/mmol and an eGFR 30–44 mL/min/1.73 m^2^ or 3.5-35 mg/mmol and an eGFR of 30–60 mL/min/1.73 m^2^) of the KHA Risk Matrix in this part of the analysis. Using the green/normal risk category in the KHA Risk Matrix as the reference category, multinomial logistic regression was used to calculate odds ratios (ORs) and 95 % CIs of progression in the KHA Risk Matrix to the yellow zone (3.5-35 mg/mmol) or the red zone (>35 mg/mmol) associated with baseline characteristics including UTI, hypertension, alcohol consumption, current smoking, diabetes, BMI and WHR. Only the multinomial logistic regression models adjusted for baseline ACR, age, gender and community are presented. Mutual adjustment for baseline diabetes, WHR and hypertension was used to assess whether these factors were independently associated with risk. All statistical analyses were performed using the SAS software package, version 9.4 (SAS Institute, Cary, NC).

## Results

### Risk associated with abnormal baseline albuminuria

A total of 623 people were followed for a total of 6009 person-years. Baseline characteristics of the participants from the three central Australian communities according to baseline ACR levels are shown in Table 1. Of the participants who had a baseline ACR reading, 69.3 % (*n* = 413) had normal ACR levels and 30.7 % (*n* = 180) of participants had an ACR of ≥3.5 or higher levels. There were a total of 30 participants who did not have a baseline ACR measurement. These participants had similar characteristics to the participants with an ACR ≥3.5 with the exception that were fewer diabetics; had lower median BMI; less hypertension and fewer current smokers.

**Table 1:** Baseline characteristics for 623 central Australian cohort participants stratified by baseline ACR level

	All participants	ACR <3.5 mg/mmol	ACR ≥3.5 mg/mmol
	*(n = 623)*	*(n = 413)* ^a^	*(n = 180)* ^a^
Age at recruitment	30 (15–85)	28 (15–79)	35 (16–85)
Age at end of follow-up	40 (19–90)	38 (19–89)	45 (27–90)
Years of follow-up	9.8 (0.5-10.8)	9.8 (0.9-10.8)	9.8 (0.5-10.8)
Person-years of follow up	6009	4001	1734
Male gender	267 (42.9)	189 (45.8)	67 (37.2)
Diabetes at baseline	79 (12.7)	28 (6.8)	47 (26.1)
Fasting glucose, mmol/L	4.4 (2.7-19.8)	4.3 (2.7-17.2)	4.7 (3.2-19.8)
BMI, kg/m^b^	25.2 (13.4-51.1)	24.4 (13.4-48.4)	26.6 (15.5-51.1)
WHR	0.87 (0.70-1.33)	0.86 (0.70-1.33)	0.89 (0.73-1.20)
Alcohol abstinence	395 (62.3)	244 (40.26)	132 (66.3)
Current smoker	177 (29.2)	131 (32.3)	42 (24.1)
Hypertension	127 (20.4)	57 (13.8)	64 (35.6)
Systolic BP, mmHg	125 (93–198)	124 (95–174)	130 (93–198)
Diastolic BP, mmHg	72 (44–119)	70 (44–105)	75 (51–117)
ACEi use	1 (0.2)	1 (0.2)	0
Other BP medication use	13 (2.1)	6 (1.5)	6 (3.3)
Homocysteine, μmol/L	13.5 (4.0-46.1)	13.2 (4.0-46.1)	14.1 (5.9-41.2)
Prevalent UTI	42 (6.7)	26 (6.3)	15 (8.3)
Prevalent haematuria			
Nil	442 (70.9)	303 (73.4)	111 (61.7)
Trace	70 (11.2)	48 (11.6)	22 (12.2)
Small	42 (6.7)	25 (6.1)	17 (9.4)
Moderate	30 (4.8)	21 (5.1)	8 (4.4)
Large	39 (6.3)	16 (3.9)	22 (12.2)
KHA Matrix Green Zone^b^	201 (64.4)	201 (100.0)	0
KHA Matrix Yellow Zone^b^	82 (26.3)	0	82 (73.9)
KHA Matrix Red Zone^b^	29 (9.3)	0	29 (26.1)

^a^Excludes 30 participants without a baseline ACR measurement; ^b^Based upon baseline ACR measurement among participants with 1 or more ACR measurements during follow-up (*n* = 312). Continuous data are median (range), categorical data are prevalence and presented as n (%). *BMI* Body mass index, *WHR* Waist hip ratio, *ACEi* Angiotensin-converting-enzyme inhibitor, *ACR* Albumin creatinine ratio, *BP* Blood pressure, *UTI* Urinary tract infection, *KHA* Kidney Health Australia

Among the 623 participants, a total of 16 incident cases of ESKD were observed, of whom all but three were given dialysis (Table 2). The overall standardised incidence rate (SIR) for ESKD was 340/100,000 person-years, this being significantly higher among participants with elevated baseline ACR levels. Likewise, SIRs for dialysis, CVD, chronic disease mortality, all-cause mortality (including and excluding traumatic deaths) were all significantly higher among participants with elevated baseline ACR. Of the four deaths attributed to renal failure, three were among participants with abnormal baseline albuminuria.

**Table 2:** Standardised^1^ incidence rates for ESKD, dialysis, CVD and mortality

	All participants			ACR <3.5 mg/mmol ^d^			ACR ≥3.5 mg/mmol ^d^		
Outcome	Events	Person-years^b^	Incidence^c^ (95%CI)	Events	Person-years^b^	Incidence^c^ (95%CI)	Events	Person-years^b^	Incidence (95%CI)
Diagnosis									
ESKD	16	5,961	340 (334–346)	2	4,001	70 (67–73)	12	1,691	789 (774–809)
Dialysis	13	5,976	212 (202–222)	1	4,000	26 (22–31)	10	1,706	512 (484–539)
CVD	67	5,769	1,060 (1037–1083)	33	3,909	868 (841–895)	31	1,604	1437 (1391–1483)
Mortality									
Renal death	4	6,009	61 (60–62)	0	4,001	-	3	1,734	125 (122–128)
All chronic disease deaths combined^e^	19	6,009	283 (276–290)	8	4,001	203 (196–211)	9	1,734	364 (351–377)
Non-traumatic death^f^	40	6,009	617 (603–631)	17	4,002	446 (430–461)	17	1,734	746 (721–772)
All-cause mortality	53	6,009	860 (839–880)	28	4,002	730 (707–754)	18	1,734	904 (867–941)

^a^The standard population used is the non-diabetic cohort members; ^b^person-years = time from recruitment until incident diagnosis or end of follow up period and rounded to nearest full number; ^c^per 100,000 person-years, showing age and gender adjusted standardised incidence rate; ^d^excludes 30 participants without a baseline ACR measurement; ^e^CVD, renal, cancer or diabetic cause of death; ^f^excluding a traumatic cause of death. NB: A total of 53 participants died during the follow-up period (all-cause mortality), of which 40 were considered non-traumatic deaths (that is, not including deaths such as motor vehicle accidents and homicides). Of the non-traumatic deaths, any underlying cause of death attributed to a chronic disease, including CVD, renal disease, cancer or diabetes was considered a chronic disease death (*n* = 19). Of the chronic disease attributed deaths, four had renal underlying causes recorded. The difference between the numbers of all participants and the ACR categories is due to the 30 participants without a baseline ACR measurement. *ESKD* End stage kidney disease, *CVD* Cardiovascular disease, *ACR* Albumin creatinine ratio

Cox’s proportional hazards models showed that an elevated baseline ACR was associated with an almost 11-fold risk of ESKD and a 17-fold risk of dialysis after adjustment for age and gender (Table 3; Model 1). Only a borderline risk association of elevated ACR was observed with CVD and no association was observed with mortality from all-causes and chronic diseases. Controlling for BMI made little difference to the strength of the association of an abnormal baseline ACR with ESKD (HR = 11.5 [95 % CI 2.44-54.0], *p*-value = 0.003) and dialysis (HR = 18.4 [95 % CI 2.28-148], *p*-value = 0.006). Further adjustment for baseline WHR did not alter the association with ESKD and dialysis (data not shown). These effects were slightly attenuated with adjustment for community of residence (Model 2). The final model (Model 3) included the further adjustment of blood pressure medication at baseline and fasting glucose. Model 3 showed a slightly attenuated risk estimate in comparison to Model 2; however, the strength of the association of ESKD and dialysis with ACR remained very strong.

**Table 3:** Hazard ratio associated with ACR ≥3.5^1^ for ESKD, dialysis, CVD and mortality

		Model 1^b^			Model 2 ^c^			Model 3 ^d^		
Outcome	Events	HR	95 % CI	*P*-value	HR	95 % CI	*P*-value	HR	95 % CI	*P*-value
Clinical Diagnosis										
ESKD	12	10.7	(2.31-49.1)	*0.002*	9.55	(2.08-43.9)	*0.004*	7.60	(1.42-40.6)	*0.018*
Dialysis	10	17.4	(2.16-139)	*0.007*	15.2	(1.89-121)	*0.010*	13.0	(1.41-119)	*0.024*
CVD	31	1.79	(1.08-2.97)	*0.024*	1.66	(1.00-2.75)	*0.052*	1.23	(0.71-2.13)	*0.462*
Mortality										
All chronic disease attributed deaths combined^e^	9	2.00	(0.72-5.52)	*0.182*	1.99	(0.71-5.53)	*0.189*	2.39	(0.76-7.49)	*0.136*
Non-traumatic^f^	17	1.59	(0.77-3.29)	*0.215*	1.53	(0.73-3.19)	*0.262*	1.94	(0.85-4.42)	*0.117*
All-cause mortality	18	1.12	(0.58-2.15)	*0.739*	1.11	(0.57-2.16)	*0.751*	1.40	(0.68-2.90)	*0.365*

^a^ACR < 3.5 mg/mmol as reference category and excludes 31 participants without an ACR measurement at baseline recruitment; ^b^Model 1 adjusted for gender and age at baseline recruitment; ^c^Model 2 adjusted for gender, age at baseline recruitment and community; ^d^Model 3 adjusted for gender, age at baseline recruitment, community, and further adjusted for baseline blood pressure medication and fasting glucose at baseline; ^e^Any underlying cause of death attributed to a chronic disease, including CVD, renal disease, cancer or diabetes; ^f^All deaths excluding a traumatic cause of death (such as a motor vehicle accidents). *ESKD* End stage kidney disease, *CVD* Cardiovascular disease, *ACR* Albumin creatinine ratio

### Progression of albuminuria

A total of 277 participants had a baseline reading of ACR <35 mg/mmol, at least one ACR follow-up screening and were not prescribed blood pressure medication. Overall, this subpopulation was generally similar characteristics to the overall analytical cohort with a slightly higher prevalence of diabetes at baseline (Table 4). Participants who were observed to have an increase in albuminuria were generally more likely to be female and had a higher proportion of baseline diabetes and hypertension. The mean follow-up time from baseline recruitment to follow-up ACR measurement for participants who remained in the KHA green zone (normal risk category) was 9.3 years, 8.7 years for participants that progressed to the KHA yellow zone (mildly increased risk category) and 7.8 years for participants who progressed to the KHA red zone (very high increased risk category).

**Table 4:** – Characteristics^a^ of 277 participants with a baseline reading of ACR ≤35 mg/mmol^2^ by follow-up ACR^c^

	All participants	Green/normal risk category (ACR <3.5)	Yellow/mild risk category (ACR 3.5-35)	Red/high risk category (ACR >35)
	*(n = 277)*	*(n = 139)*	*(n = 93)*	*(n = 45)*
Age at recruitment	31 (15–74)	29 (16–70)	33 (15–70)	30 (16–74)
Age at exit	40 (19–80)	40 (19–75)	41 (22–80)	40 (23–80)
Male gender	127 (45.9)	66 (47.5)	45 (48.4)	16 (35.6)
Alcohol abstinence	166 (61.3)	88 (63.8)	48 (53.3)	30 (69.8)
Current smoker	78 (28.9)	39 (28.3)	28 (31.5)	11 (25.6)
Diabetes	42 (15.2)	9 (6.5)	18 (19.4)	12 (33.3)
BMI, kg/m^b^	25.8 (14.6-48.4)	24.8 (14.6-44.1)	26.4 (16.7-46.2)	26.9 (19.6-48.4)
WHR	0.87 (0.70-1.33)	0.85 (0.70-1.04)	0.89 (0.70-1.33)	0.91 (0.78-1.03)
Hypertension	53 (19.1)	25 (18.0)	17 (18.3)	11 (24.4)
Systolic BP, mmHg	125 (93–184)	124 (93–134)	125 (96–184)	127 (103–164)
Diastolic BP, mmHg	71 (44–108)	71 (45–108)	70 (44–105)	72 (53–100)
Homocysteine, μM	13.8 (4.0-46.1)	13.2 (6.3-46.1)	14.6 (7.0-37.9)	12.4 (3.4-41.1)
Prevalent UTI	14 (5.1)	7 (5.0)	4 (4.3)	3 (6.7)

^a^Continuous data are median (range), categorical data are prevalence, presented as n (%);^b^ with at least one ACR measurement during follow-up and were not users of blood pressure medication (including ACEi); ^c^ according to KHA Risk Matrix zones – green/normal risk category, yellow/mildly increased risk category and red/very high increased risk category. *BMI* Body mass index, *WHR* Waist hip ratio, *ACR* Albumin creatinine ratio, *BP* Blood pressure, *UTI* Urinary tract infection

**Fig. 1:**
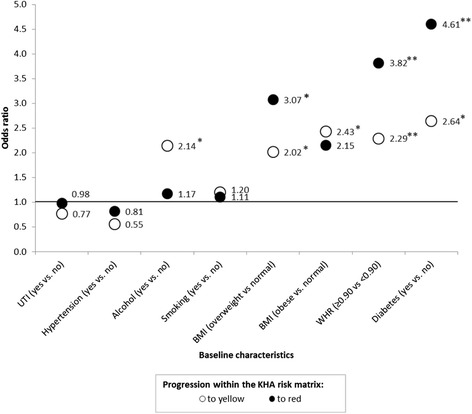
Baseline predictors of progression in the KHA Risk Matrix Legend: The odds of progression to yellow (3.5 – 35 mg/mmol) and to red (>35 mg/mmol) as described in the Kidney Health Australia, clinical guidelines [7] and the Central Australian Rural Practitioners Association (CARPA) Standard Treatment guidelines [11]. All models adjusted for age, gender, community and ACR at baseline. *Pvalue <0.05; **Pvalue ≤ 0.01. 95 % Confidence intervals and *P*values are shown in Additional file 1: Table S1

Multinomial logistic regression models adjusted for baseline ACR, age, gender and community showed that baseline diabetes was associated with a more than two-fold increased risk of progression to the yellow zone of the KHA Risk Matrix and more than four-fold risk of progression to the red zone (very high increased risk) from the green zone (normal risk) (Fig. 1 and Additional file 1: Table S1). Diabetes was independently associated with risk of a strong increase in albuminuria even after further adjustment for baseline hypertension and WHR (from green to yellow OR = 2.51 [95 % CI 0.94-6.68], *p*-value = 0.06; from green to red OR = 3.76 [95 % CI 1.17-12.08], *p*-value = 0.03). Abdominal obesity was also associated with a two-fold increased risk of progression of ACR levels between 3.5-35 mg/mmol and almost four-fold risk of progression of ACR levels >35 mg/mmol. Further adjustment for baseline diabetes, baseline ACR levels and hypertension only slightly attenuated the association with WHR (from green to yellow OR = 2.13 [95 % CI 1.12-4.04], *p*-value = 0.02; from green to red OR = 3.26 [95 % CI 1.36-7.82], *p*-value = 0.008). Smoking, baseline UTI, or hypertension were not significantly associated with progression in the KHA Risk Matrix. A BMI >25 kg/m^2^ was generally associated with a significantly increased odds of progression in the KHA Risk Matrix. Alcohol consumption was associated with a moderate progression in ACR level, however alcohol consumption was not associated with follow-up ACR levels greater than 35 mg/mmol. Further adjustment for the respective models that included hypertension (both diastolic and systolic), UTI, haematuria, homocysteine, smoking, and alcohol consumption was not informative and did not substantially change the results (data not shown).

## Discussion

In this central Australian Aboriginal community-based cohort, a single abnormal albuminuria reading was independently associated with a strongly increased risk of ESKD, dialysis and possibly CVD outcomes over the subsequent 10 years. Given the high burden of CKD in remote Aboriginal communities and the strong risk association of a single abnormal ACR level with future ESKD and dialysis outcomes, these results warrant an appropriate clinical response to an abnormal spot ACR assessment. Diabetes and a WHR ≥0.90 were both independently associated with risk of progression in ACR levels, suggesting that including abdominal adiposity as an early target for CKD prevention strategies may merit investigation. We have therefore identified clinical predictors of a significant progression in ACR levels that can also be readily measured in remote Australian PHC settings.

An abnormal albuminuria level (i.e. an ACR of ≥3.5 or higher) has been consistently reported to be associated with subsequent renal disease, CVD and natural death [22] in other populations [23–26] including the Tiwi community [3, 5]. Therefore the association of a single baseline reading predicting ESKD and dialysis outcomes is not new. However, the strength of the association is striking even after controlling for baseline UTI, diabetes and obesity. CKD and ESKD are thought to be caused by persistent low birth weights (low number of nephrons [8]), maternal and infant malnutrition, repeated and persistent infections and inflammation that damage the kidneys, and changes to the exercise and dietary patterns related to “Westernization” [5, 8]. Other factors that contribute to the excess kidney disease levels in this group are a high prevalence of diabetes, smoking and poor diet and inadequate access to basic health hardware, a healthy food supply and other preventive services [5, 8]. The current data support the clinical guidelines developed specifically for this population group in terms of renal disease screening approaches including the use of the KHA Risk Matrix [15]. To our knowledge, this is the first time the ACR component of the KHA Risk Matrix has been used to evaluate CKD risk among Aboriginal people in a remote setting.

Within this population who live with the burden of a high incidence of ESKD, CVD and mortality, in terms of interventions, a spot check for an ACR measurement of ≥3.5 mg/mmol is a potentially cost effective method applicable to the remote setting. It presents itself as a candidate able to assess future risk of ESKD and CVD which could lead to better directed prevention strategies being initiated at a stage earlier in the disease process. While current guidelines for CKD diagnosis involve two eGFR or ACR readings at least three months apart, a single ACR measurement, as recommended elsewhere [11, 15], may be an effective surrogate. We propose not a tool for diagnosing CKD but a tool to screen, within a population at high risk, people who could benefit from earlier CKD prevention strategies. The present results strongly support the use of a single ACR reading, as it is already within the annual Medicare adult health check for Aboriginal and Torres Strait Islander people. Using a single ACR of ≥3.5 mg/mmol as a means to identify people at risk of ESKD may result in a possible excess in the clinical classification of at-risk due to the association of prevalent UTI and ACR. However, we propose that the inclusion of people over-classified as ‘at risk’ would be preferable than the current under classification and under diagnosis of CKD in remote communities.

Surprisingly, we observed no association between hypertension and progression within the KHA Risk Matrix which is counter to established risk factors for ESKD [27]. Within this statistical model we controlled for the use of baseline hypertensive medication by excluding participants using baseline hypertensive medication from the model and the median systolic and diastolic blood pressure were within normal ranges. We were unable to disentangle the relationship between blood pressure and ACR; specifically whether an increase in ACR precedes hypertension or vice versa. It is possible that albuminuria and hypertension share a common and parallel aetiology, with both conditions reflecting vascular damage from inflammatory processes secondary to oxidative stress of the endothelium and sub-endothelial tissues [19]. The lack of an association of hypertension and progression within the KHA Risk Matrix may also be a chance finding.

With the number of prevalent cases of Aboriginal and Torres Strait Islander People with treated end-stage kidney disease increasing [28] along with advances to treatment and sustainment of life, the unsustainable cost of renal failure is not only a health service provision and health care costing issue, but also the loss of community members who opt out of receiving treatment and premature death and/or relocating to larger towns for renal replacement therapy. Interventions to reduce the risk of CKD will only be sustainable if they are applied over the life-course and address underlying socio-economic conditions and social determinants of health [4]. Targeted interventions include improving the health of women before and during pregnancy with nutrition and spacing of births, reducing smoke and alcohol exposure in utero and management of diabetes in pregnancy, avoiding under nutrition and preventing obesity in infancy and childhood and ensuring adequate public health measures to avoid repeated infections [4]. Furthermore, the monitoring of albuminuria and treating hypertension with renoprotective therapy along with aggressive management of diabetes have been shown to reduce renal failure, cardiovascular disease and mortality in a Australian Aboriginal community [29, 30]. ACR is amenable to point of care testing in the remote primary health care setting [9]. Although economic evaluation was outside the scope of this study, others have found the screening cost incurred by reducing the number of people with CKD progressing into the high-cost treatment of ESKD would be a more sustainable solution [10].

There are limitations to the current analysis and its findings reported here. Our observations are from central Australia and further investigation is required to assess the applicability to other Aboriginal and Torres Strait Islander populations particularly given the variability of other characteristics such as environment and socioeconomic factors affecting risk profiles [31]. However, as an intermediate indicator between environmental drivers of risk and ESKD, a spot ACR is a potentially generalizable screening tool. Furthermore this study adds to a growing evidence base among Aboriginal and Torres Strait Islander populations for the role of albuminuria >3.5 mg/mmol as a marker of early CKD [3, 5]. We were unable to expand the combined CVD endpoint according to PVD, stroke, CHD and CHF outcomes. This may explain the borderline significance of CVD. However, in a recent study an ACR cut off of ≥ 5.7 mg/mmol was associated with more than a 2-fold increased odds of CVD [17], so the current cut off of ≥ 3.5 mg/mmol used in this analysis may have been too low to capture the CVD risk association. Further, we understand that these diseases do not necessarily have the same aetiology and may be associated with ACR differently. In addition, among earlier cases of ESKD and CVD, ICD-9 codes were used for the diagnosis of renal and CVD outcomes. This may have led to an under-diagnosis and could have biased the results towards the null. Previous studies have reported substantial intra-individual variability of ACR [32] from day-to-day spot urine samples and this could be a source of error. However, given the strong association with ESKD and baseline ACR in this population, a single spot check as a diagnostic tool warrants further investigation, and has been shown to be robust measure of renal disease [10] including among remote Aboriginal populations [3]. This analysis was not a screening and diagnostic study therefore sensitivity and specificity were not examined. Furthermore, participants with ACR measurements during their follow-up tended to be less healthy than participants who only had an ACR at baseline measurement. The use of eGFR as per the screening protocol as recommended by KHA also requires investigation, because ACR and eGFR are not always associated in the same way as in non-Indigenous Australians: in an urban setting a high prevalence of microalbuminuria but not low eGFR was observed in an Indigenous population [33].

## Conclusions

Baseline albuminuria was a strong predictor of risks of ESKD and dialysis in this population. For clinical identification of individuals at risk of future ESKD, a single spot urine ACR analysis may be a useful and efficient strategy to screen for future CKD risk among remote communities. Targeting individuals with diabetes and high central obesity to avoid increases in albuminuria may also prevent future CKD and CVD complications. This research provides evidence that the KHA Risk Matrix classification applied in the PHC setting is useful in identifying Aboriginal persons at elevated risk of CKD.

## Abbreviations

95 % CI, 95 % confidence interval; ACEi, angiotensin-converting-enzyme inhibitor; ACR, albumin creatinine ratio; BMI, body mass index; BP, blood pressure; CARPA, Central Australian Rural Practitioners Association; CHD, chronic heart disease; CHF, congestive heart failure; CKD, chronic kidney disease; CVD, cardio-vascular disease; eGFR, estimated glomerular filtration rate; ESKD, end stage kidney disease; HR, hazard ratio; ICD 10, International Classification of Disease 10th Edition; ICD 9, International Classification of Disease 9th Edition; KHA, Kidney Health Australia; OR, odds ratio; PHC, primary health care; PVD, pulmonary vascular disease; SMR, standardised mortality ratio; UTI, urinary tract infection; WHR, waist to hip ratio

## Additional file

Additional file 1: Table S1. Odds of progression^1^ within the Kidney Health Australia Risk Matrix. (DOCX 17 kb)
