# Supplementary material for: Clinical outcomes associated with albuminuria in central Australia: a cohort study
Source: BMC Nephrol. 2016 Aug 5;17:113. doi: 10.1186/s12882-016-0328-1 (PMC4974695; doi:10.1186/s12882-016-0328-1)
Supplement: Additional file 1: Table S1. — Odds of progression1 within the Kidney Health Australia Risk Matrix. (DOCX 17 kb) [file 12882_2016_328_MOESM1_ESM.docx]

### Additional table 1 – Odds of progression within the Kidney Health Australia Risk Matrix

|  | **Odds Ratio** | **95% CI** | |  |
| --- | --- | --- | --- | --- |
|  |  | **Lower** | **Upper** | ***p_value_*** |
| **UTI (yes vs. no)** |  | | |  |
| Green | Reference | | |  |
| Yellow | 0.77 | 0.19 | 3.21 | 0.72 |
| Red | 0.98 | 0.17 | 5.50 | 0.98 |
| **Hypertension (yes vs. no)** |  | | |  |
| Green | Reference | | |  |
| Yellow | 0.55 | 0.24 | 1.28 | 0.17 |
| Red | 0.81 | 0.27 | 2.42 | 0.71 |
| **Alcohol (yes vs. no)** |  |  |  |  |
| Green | Reference |  |  |  |
| Yellow | 2.14 | 1.05 | 4.38 | 0.04 |
| Red | 1.17 | 0.43 | 3.18 | 0.76 |
| **Smoking (yes vs. no)** |  |  |  |  |
| Green | Reference | | |  |
| Yellow | 1.20 | 0.59 | 2.44 | 0.61 |
| Red | 1.11 | 0.40 | 3.10 | 0.85 |
| **BMI (overweight vs normal)** |  |  |  |  |
| Green | Reference | | |  |
| Yellow | 2.02 | 1.04 | 3.93 | 0.04 |
| Red | 3.07 | 1.20 | 7.90 | 0.02 |
| **BMI (obese vs. normal)** |  |  |  |  |
| Green | Reference | | |  |
| Yellow | 2.43 | 1.05 | 5.62 | 0.04 |
| Red | 2.15 | 0.69 | 6.77 | 0.19 |
| **WHR > 90** |  |  |  |  |
| Green | Reference | | |  |
| Yellow | 2.29 | 1.22 | 4.30 | 0.01 |
| Red | 3.82 | 1.62 | 8.98 | 0.002 |
| **Diabetes (yes vs. no)** |  |  |  |  |
| Green | Reference | | |  |
| Yellow | 2.64 | 1.04 | 6.71 | 0.04 |
| Red | 4.61 | 1.52 | 13.97 | 0.007 |

The odds of progression from green to yellow (3.5 – 35 mg/mmol) and green to red (≥35mg/mmol) as described in the Kidney Health Australia [[7](#_ENREF_7)], clinical guidelines The Central Australian Rural Practitioners Association (CARPA) Treatment guidelines [[11](#_ENREF_11)] using multinomial logistic regression. All models adjusted for age, gender, community and ACR at baseline.
